# Long-Term Outcomes of Concomitant Left-Sided Cryoablation During Totally Endoscopic Mitral Valve Surgery

**DOI:** 10.1093/icvts/ivaf217

**Published:** 2025-09-27

**Authors:** Ilia Bazhanov, Johannes Petersen, Yalin Yildirim, Jonas Pausch, Evaldas Girdauskas, Yousuf Al Assar, Hermann Reichenspurner, Simon Pecha

**Affiliations:** Department of Cardiovascular Surgery, University Heart and Vascular Center Hamburg, University Medical-Center Hamburg-Eppendorf, Hamburg 20246, Germany; Department of Cardiovascular Surgery, University Heart and Vascular Center Hamburg, University Medical-Center Hamburg-Eppendorf, Hamburg 20246, Germany; DZHK (German Centre for Cardiovascular Research), Partner Site Hamburg/Kiel/Lübeck, Hamburg 20246, Germany; Department of Cardiovascular Surgery, University Heart and Vascular Center Hamburg, University Medical-Center Hamburg-Eppendorf, Hamburg 20246, Germany; Department of Cardiovascular Surgery, University Heart and Vascular Center Hamburg, University Medical-Center Hamburg-Eppendorf, Hamburg 20246, Germany; Department of Cardiothoracic Surgery, University Hospital Augsburg, Augsburg 86156, Germany; Department of Cardiovascular Surgery, University Heart and Vascular Center Hamburg, University Medical-Center Hamburg-Eppendorf, Hamburg 20246, Germany; Department of Cardiovascular Surgery, University Heart and Vascular Center Hamburg, University Medical-Center Hamburg-Eppendorf, Hamburg 20246, Germany; DZHK (German Centre for Cardiovascular Research), Partner Site Hamburg/Kiel/Lübeck, Hamburg 20246, Germany; Department of Cardiovascular Surgery, University Heart and Vascular Center Hamburg, University Medical-Center Hamburg-Eppendorf, Hamburg 20246, Germany; DZHK (German Centre for Cardiovascular Research), Partner Site Hamburg/Kiel/Lübeck, Hamburg 20246, Germany

**Keywords:** atrial fibrillation, ablation, cryoablation, mitral valve surgery, endoscopic mitral valve surgery, minimally invasive cardiac surgery

## Abstract

**Objectives:**

Concomitant atrial fibrillation ablation is a well-established procedure in patients undergoing mitral valve (MV) surgery. However, data concerning the long-term outcomes of cryoablation performed during totally endoscopic MV surgery remain limited. Furthermore, different lesion sets and energy sources used in endoscopic approach may result in varying outcomes. We therefore, analysed rhythm outcome in patients undergoing left-sided cryoablation during totally endoscopic MV surgery.

**Methods:**

Patients, who underwent totally endoscopic MV surgery with concomitant left-sided cryoablation between 2016 and 2023 at our centre were included in the study. The retrospective data analysis was based on 24-h Holter monitor follow-up data.

**Results:**

A total of 123 patients were included in the study. No complications related to the ablation procedure were observed. The median follow-up period was 36.0 (interquartile range: 17-60) months. During this period, 34 episodes of atrial fibrillation recurrence were documented, corresponding to a recurrence rate of 8.43 per 100 patient-years (95% confidence interval: 5.90-11.73). The estimated freedom from AF at 1, 3, and 5 years were 96.6%, 86.3%, and 69.4% respectively. Type of atrial fibrillation (*P* = .004; hazard ratio [HR]: 2.521; 95% confidence interval [CI]: 1.347-4.716) and left atrial volume (*P* = .003; HR: 1.010; 95% CI: 1.003-1.016) were identified as predictors for atrial fibrillation recurrence.

**Conclusions:**

Concomitant left-sided cryoablation during totally endoscopic MV surgery is a safe and effective procedure for atrial fibrillation treatment. The encouraging long-term outcomes support the consideration of this approach in totally endoscopic mitral surgery.

## Graphical abstract

**Figure:**
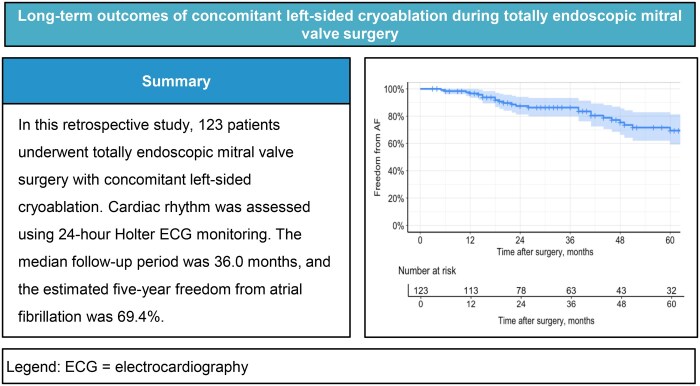

## INTRODUCTION

Atrial fibrillation (AF) has been identified as the most prevalent clinically significant cardiac arrhythmia, affecting between 1.9% and 2.9% of the European population with projections suggesting this number will double by 2060.^1^^,^^2^ AF is associated with an elevated risk of cardiovascular incidents, resulting in a significant increase in healthcare costs.^3^ Especially patients with mitral valve (MV) disease have a high incidence of AF.^4^ The Maze procedure, first described by James Cox in 1987, represented a major breakthrough in the surgical treatment of AF.^5^ Initially performed using the “cut-and-sew” technique, it has since evolved to the Cox-maze IV procedure, which utilizes less invasive methods employing radiofrequency and cryoenergy. This has enhanced safety and simplified the procedure, leading to its wider adoption.^6^ Concomitant AF ablation during cardiac surgery is now recognized as an effective method and recommended in the current guidelines as an Ia indication.^7^^,^^8^ While bipolar radiofrequency ablation is predominantly used for epicardial ablation, cryoenergy has proven effective in achieving transmurality during endocardial ablation, making it frequently utilized in MV surgeries (MVS).^9–12^ The utilization of minimally invasive access with a totally endoscopic approach via a right thoracotomy is a procedure of increasing prevalence, particularly in MV procedures.^13–15^ However, it should be taken into consideration that during endoscopic procedures, the visualization of the operative field is entirely different, which may affect the outcomes. Meanwhile, the results of concomitant left-sided cryoablation during these procedures are still limited. Therefore, the aim of our study was to analyse the single centre experience and long-term outcomes of concomitant left-sided cryoablation during totally endoscopic MVS.

## METHODS

### Ethical statement

This retrospective single-centre study was established in accordance with the ethical standards of the Declaration of Helsinki (1964) and approved by the local ethics committee on 12th December 2022 (No. 2020-10183). Due to the retrospective study design, written informed patient consent was waived. Any collection and storage of data from research participants for multiple and indefinite use was conducted in accordance with the WMA Declaration of Taipei. The research ethics committee approved the establishment and ongoing use of such databases.

### Study cohort

Patients who underwent totally endoscopic MV surgery with concomitant left-sided cryoablation at the University Heart and Vascular Center Hamburg between May 2016 and May 2023, and who had a 24-h Holter electrocardiogram rhythm assessment, were included in this retrospective study and analysed.

### Follow-up

Data were collected via a variety of methods, including standardized written questionnaires, telephone interviews, clinical visits, and patient record reviews. This comprised the current rhythm status obtained through 24-h Holter ECG or implanted cardiac rhythm devices, the need for postoperative electrical cardioversion (eCV) and/or additional catheter ablation, need for reoperation, history of stroke, and current anticoagulation therapy. Rhythm at discharge was documented by 12-lead ECG. The definition of ablation success was as follows: the absence of AF episodes longer than 30 s, as determined by 24-h Holter ECG or cardiac device interrogation following the 3-month blanking period.

### Surgical technique

Minimally invasive MVS was performed via a right anterolateral mini-thoracotomy guided by 3D endoscopy. Peripheral cardiopulmonary bypass (CPB) was established through percutaneous cannulation of the femoral vessels. MV repair or replacement was conducted using appropriate surgical techniques. In cases of severe tricuspid regurgitation, additional tricuspid valve repair (TVR) was performed using an annuloplasty. Left atrial cryoablation included a left atrial box lesion, mitral isthmus ablation, and a line to left atrial appendage (LAA). Cryoenergy was utilized as the energy source for all cases of ablation, administered through the CryoICE Cryoablation probe (Atricure, Inc., West Chester, OH) or Cardioblate CryoFlex probe (Medtronic, Inc., Minneapolis, MN). The AtriClip Pro device (AtriCure, OH, United States) was utilized for the closure of the LAA in all patients, with intraoperative transoesophageal echocardiography evaluation of successful closure.

### Statistical analysis

This was a retrospective single-centre study. Statistical analysis was performed using SPSS software (IBM Corp., Version 29.0.2.0 Armonk, NY). Continuous variables are shown as medians (25th percentile-75th percentile) and compared using the Mann-Whitney *U*-test. Categorical variables were presented as frequencies and percentages, with comparisons conducted using the chi-squared test, or Fisher’s exact test when expected cell frequencies were low. A *P*-value of <.05 was considered statistically significant. The Kaplan-Meier analysis was conducted to estimate the freedom from AF and to visually depict the recurrence of AF over time. A multivariable analysis using a stepwise Cox regression model was conducted to identify predictors of AF freedom. The selection of these variables was determined by their clinical relevance, including age, gender, left atrium (LA) volume, AF type and duration, in addition to other factors hypothesized to influence the risk of AF. The primary end-point of this study was freedom from AF during long-term follow-up.

## RESULTS

### Patient’s characteristics

A total of 710 patients underwent totally endoscopic MVS at the University Heart and Vascular Center Hamburg in the period between May 2016 and May 2023. Of these patients, 154 underwent concomitant left-sided cryoablation. A retrospective analysis was performed on 123 patients with a 24-h Holter ECG rhythm status available during follow-up. The median age of patients age was 66.0 (58.0-71.0) years, with 56.9% of patients being male. The median left ventricular ejection fraction (LVEF) was 55.0 (50.0-60.0)%, whereas the median LA volume was 95.0 (74.4-125.0) ml. Preoperative paroxysmal AF was observed in 43.1% of patients, while 38.2% exhibited persistent AF and 18.7% long-standing persistent AF. The median duration of AF was 13.0 (3.0-55.0) months. Furthermore, 5 patients (4.1%) had a history of a previous catheter ablation, while 10 patients (8.1%) had permanent pacemaker (PPM) implanted before surgery. A comprehensive overview of the patients’ baseline characteristics is provided in **Table 1**.

**Table 1.** Preoperative Baseline Characteristics

	Study cohort (*n* = 123)
Age, years	66.0 (58-71)
Male sex, *n* (%)	70 (56.9%)
BMI, kg/m²	24.86 (22.7-27.3)
AF duration, months	13 (3.0-55.0)
Type of AF	
Paroxysmal AF, *n* (%)	53 (43.1%)
Persistent AF, *n* (%)	47 (38.2%)
Long-standing persistent AF, *n* (%)	23 (18.7%)
LVEF, %	55.0 (24.0-78.0)
LA volume, mL	95.0 (74.4-125.0)
EuroScore II, %	1.54 (1.0-2.6)
Coronary artery disease, *n* (%)	20 (16.3%)
History of myocardial infarction, *n* (%)	5 (4.1%)
Previous AF catheter ablation, *n* (%)	5 (4.1%)
Thyroid disease, *n* (%)	21 (17.1%)
History of stroke, *n* (%)	7 (5.7%)
Implanted cardiac pacemaker, *n* (%)	10 (8.1%)
COPD, *n* (%)	10 (8.1%)
Diabetes mellitus, *n* (%)	4 (3.3%)
Renal insufficiency,^a^ *n* (%)	14 (11.4%)
Arterial hypertension, *n* (%)	84 (68.3%)
Hyperlipidemia, *n* (%)	37 (30.1%)

a Defined as glomerular filtration rate <60 ml/min.

Abbreviations: AF = atrial fibrillation; BMI = body mass index; COPD = chronic obstructive pulmonary disease; LVEF = left ventricfular ejection fraction.

### Procedural data

In 9 cases (7.3%), MV repair was not a viable option, and therefore replacement of the valve was performed. Furthermore, 16 patients (13.0%) exhibited concomitant significant tricuspid regurgitation, necessitating concomitant TVR. A complete left-sided cryoablation set was performed in all 123 patients (100.0%). The median procedural time was 245.0 (222.0-280.0) min. The median cross-clamp time was 95.0 (79.0-110.60) min, and the median CPB time was 162.0 (138.0-193.3) min.

### Peri- and postoperative outcome

No major complications were observed in relation to LAA occlusion and ablation. Consequently, no intra- or perioperative deaths were observed, while 4 patients (3.3%) died during follow-up. Two patients (1.6%) suffered a perioperative stroke, and 1 patient (0.8%) had a stroke during the follow-up period. Postoperative PPM was necessary in 7 patients (5.7%). Of these, 4 patients underwent concomitant TVR. During the follow-up period a PPM was needed in 3 additional patients (2.4%).

Postoperative eCV was required in 11 patients (8.9%) within the first 3 months after surgery (the blanking period), comprising: 3 patients (5.7%) with paroxysmal, 6 patients (12.8%) with persistent, and 2 patients (8.7%) with long-standing persistent AF (*P* = .494).

Anticoagulation therapy data were available for 120 patients. At the time of follow-up, more than half of the patients (56.5%) were on new oral anticoagulants therapy, 13% were receiving a vitamin K antagonist, 15.4% were taking aspirin, and 12.2% were not on any anticoagulation or antiplatelet medication. It is noteworthy that only 1 patient (0.8%) experienced a stroke during the follow-up period.

### Rhythm results

**Figure 1.**
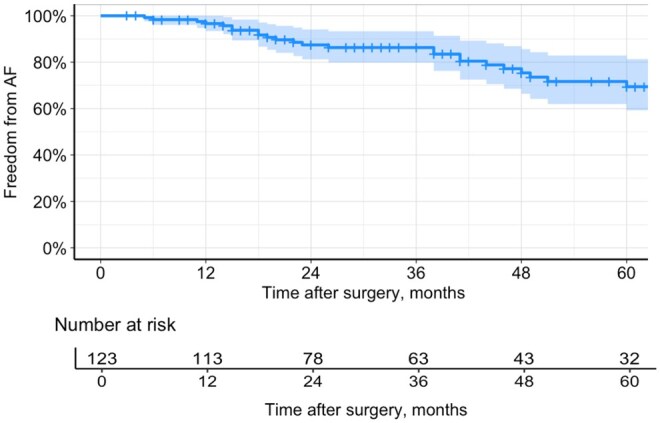
Kaplan-Meier Curve for Freedom from Atrial Fibrillation

**Figure 2.**
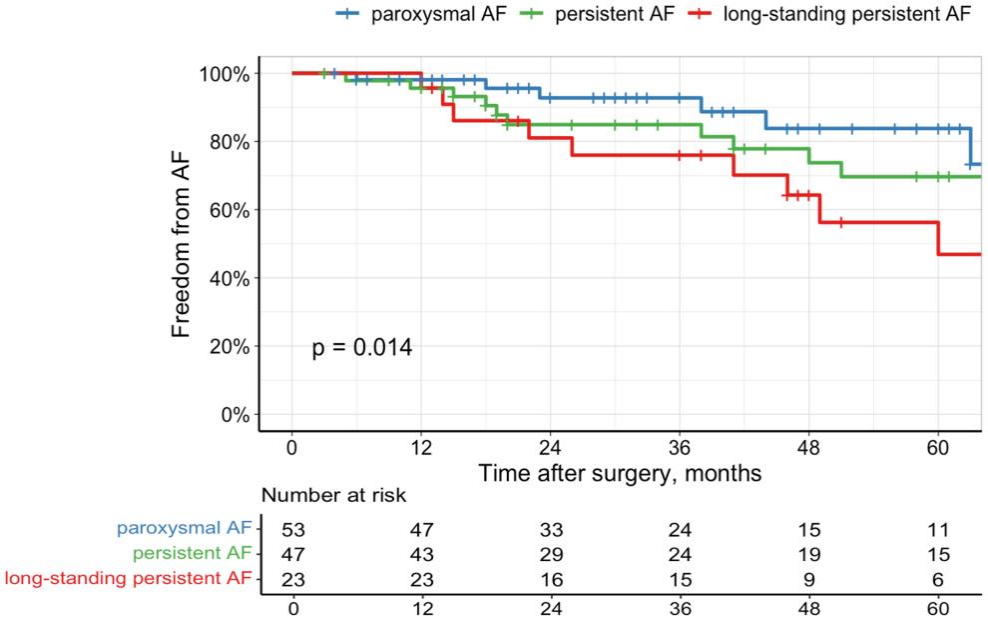
Kaplan-Meier Curve for Freedom from AF according to Pre-Operative Type of AF

A total of 80 patients (65.0%) were discharged in sinus rhythm (SR). As anticipated, the majority of discharges in SR were among patients with paroxysmal AF (42 out of 53 patients, 79.2%). In comparison, among the non-paroxysmal AF group, only 38 out of 70 patients (54.3%) were discharged in SR (*P* = .004). The median follow-up period was 36.0 (17-60) months. During this period, 34 patients experienced AF recurrence, corresponding to a recurrence rate of 8.43 per 100 patient-years (95% confidence interval [CI]: 5.90-11.73). In the overall cohort the estimated freedom from AF at 1, 3, and 5 years were 96.6% (95% CI: 93.27-99.23), 86.3% (95% CI, 79.64-92.96), and 69.4% (95% CI, 58.42-80.38) respectively (**Figure 1**). The log-rank test revealed a statistically significant difference in freedom from AF between the groups (*P* = .014). However, when comparing the groups individually, no significant difference was found between patients with paroxysmal and persistent AF (*P* = .350). Significant differences were observed between paroxysmal and long-standing persistent AF (*P* = .013), as well as between persistent and long-standing persistent AF (*P* = .032) (**Figure 2**).

### Multivariable regression analysis

Multivariable Cox regression analysis revealed type of AF (*P* = .004; hazard ratio [HR]: 2.521; 95% CI: 1.347-4.716) and left atrial volume (*P* = .003; HR: 1.010; 95% CI: 1.003-1.016) as predictors for AF recurrence.

## DISCUSSION

The development of surgical treatment of AF has come a long way; however, many contentious issues are still unresolved. One of the most debated topics is the question of the optimal ablation set strategy. Despite the fact that a randomized study published by Gillinov et al did not show a clear advantage for biatrial ablation compared to pulmonary vein isolation (PVI), numerous other studies have reported better outcomes with left-sided or biatrial ablation compared to PVI alone.^16–18^

Comparing biatrial versus left atrial ablation, the data remain controversial. While 3 meta-analyses have showed higher AF freedom at 12 months, no difference regarding the efficacy could be shown in 3 other meta-analyses.^16^^,^^19–23^ Moreover, many studies indicate a higher risk of PPM implantation following biatrial ablation, and a meta-analysis published by Cappabianca et al also reports an increase of bleeding after biatrial ablation.^16^^,^^20^^,^^24^^,^^25^ The elevated PPM rate after biatrial ablation might be related to the lines on the superior vena cava and near the tricuspid annulus. If not performed correctly, those lines may cause damage to the sinoatrial and atrioventricular nodes. On the other hand, some experts note, that the underlying cause is demasked sinus node dysfunction as a consequence of successful AF ablation, which might be the main reason.^26^

Another salient point is that the number of minimally invasive MVS is steadily increasing. In Germany, for instance, the proportion rose from 13.1% in 2004 to 60.8% in 2023.^15^^,^^27^ But in the majority of studies examining rhythm outcomes in concomitant AF ablation during MVS surgery, the procedures were primarily performed using a standard sternotomy approach.^10^^,^^24^^,^^28^^,^^29^ Therefore, data concerning the rhythm outcomes of AF ablation during minimally invasive MVS remains limited.^30^^,^^31^ At the same time, it is important to consider that visualization and surgical manipulation differ significantly in endoscopic procedures, which may influence the outcomes. A study by Yates revealed that the rates of AF freedom were comparable between sternotomy and the minimally invasive approach at long-term follow-up, extending up to 8 years. It is noteworthy that the minimally invasive technique exhibited superior outcomes at the 1- and 3-year follow-up.^32^ Other studies have shown comparable rhythm outcomes between these 2 approaches and a shorter intensive care unit and hospital stay despite longer operative time in minimally invasive group.^33^^,^^34^

At our centre, the standard approach during MVS since 2013 has been a right mini-thoracotomy. For patients diagnosed with AF, the standard strategy employed is left-sided cryoablation, encompassing a box lesion, mitral isthmus ablation, a line to LAA and closure of the LAA with an AtriClip. The median follow-up period for the study cohort was 36.0 (17-60) months, during which AF recurrence was documented in 34 patients (27.6%). The estimated freedom from AF in the overall cohort demonstrates a downwards trend, decreasing from 96.6% at 1 year to 69.4% at 5 years. Notably, we did not observe a statistically significant difference in AF duration between patients with paroxysmal and persistent AF (median 8.5 (2-30) vs 6 (2-12) months, *P* = .855). In contrast, patients with long-standing persistent AF exhibited a significantly longer median duration of AF (64 (29-84) months), with *P* < .001 when compared to each of the other groups. These findings suggest that the worse outcomes observed in long-standing persistent AF may be related to the markedly increased duration of AF in this group.

Given the study’s design, it remains uncertain whether patients continued to receive antiarrhythmic therapy during follow-up. Nevertheless, these findings may more accurately reflect real-world conditions, as patients experience diverse post-surgical care.

In the multivariable analysis, we observed that type of AF and LA volume was identified as statistically significant predictors for AF recurrence. This finding is consistent with previous studies.^24^^,^^35–37^ However, individual risk factors such as age, LVEF, diabetes mellitus, hypertension, and others did not demonstrate significant predictive value in our analysis. It is also noteworthy that AF duration was not a significant predictor in this study, which may be explained by the overall long AF duration, with a median of 13 (3-55) months. Furthermore, the presence of mitral regurgitation suggests that atrial remodelling may have initiated prior to the initial documented episode of AF.

The rationale behind our decision to adopt left-sided cryoablation as the standard lesion for AF treatment during MVS is, in part, predicated on the observed lower incidence of postoperative PPM in comparison to biatrial ablation. In the present study, a PPM was required in 7 patients (5.7%). Of these, 4 patients had concomitant TVR. Consequently, when considering solely the population of patients who underwent isolated MVS, a PPM was required in a mere 3 patients (2.6%). This rate is slightly lower than those reported in the literature, where incidences range between 3.3% and 17.3%.^25^^,^^31^^,^^32^^,^^38^

Current clinical guidelines recommend the continuation of oral anticoagulation after AF ablation based on the individual thromboembolic risk rather than the perceived rhythm outcome.^39^ Interestingly, in our study, a quarter of the patients did not receive anticoagulant therapy at the moment of follow-up and only 1 patient experienced a stroke. Our experience suggests, that many physicians discontinue anticoagulant therapy for patients with AF in a late postoperative period, often justifying this decision by the presence of a closed LAA and a stable SR. While such reasoning may seem logical, we currently lack robust data to support this practice.

### Limitations

The present study is subject to several limitations. Firstly, the study was conducted in a single centre, which may limit the generalizability of the findings. Secondly, in 20% of cases, 24-h Holter ECG results were not obtained during follow-up. These patients were therefore not included in the analysis, which could introduce potential bias. Thirdly, in some patients, AF was detected during 2-chamber pacemaker interrogation and counted as recurrence. The use of rhythm assessment methods with different sensitivities may have introduced potential bias. Fourthly, the follow-up assessments were conducted at varying time intervals, potentially leading to inconsistencies in the evaluation of outcomes. Finally, the inability to track the management and modification of risk factors for AF in patients may have had a significant impact on both short- and long-term outcomes.

## CONCLUSION

The present study demonstrates the long-term efficacy and safety of concomitant left-sided cryoablation during totally endoscopic MVS. The observed rate of AF recurrence is consistent with findings from previous research in patients undergoing a conventional approach, and the low incidence of PPM implantation underscores the advantages of left-sided cryoablation as a valuable technique in this type of surgery. However, it is important to acknowledge the limitations of the study and the necessity for further research to validate these findings. Furthermore, prospective randomized studies are required to investigate the optimal management of anticoagulation therapy following surgical ablation and LAA closure.

## Data Availability

The data underlying this article will be shared on reasonable request to the corresponding author.
